# Proximity‐Induced Novel Ferromagnetism Accompanied with Resolute Metallicity in NdNiO_3_ Heterostructure

**DOI:** 10.1002/advs.202101516

**Published:** 2021-08-11

**Authors:** Marco Caputo, Zoran Ristic, Rajendra S. Dhaka, Tanmoy Das, Zhiming Wang, Christan E. Matt, Nicholas C. Plumb, Eduardo B. Guedes, Jasmin Jandke, Muntaser Naamneh, Anna Zakharova, Marisa Medarde, Ming Shi, Luc Patthey, Joël Mesot, Cinthia Piamonteze, Milan Radović

**Affiliations:** ^1^ Photon Science Division Paul Scherrer Institute Villigen CH‐5232 Switzerland; ^2^ Institute of Condensed Matter Physics Ecole Polytechnique Fédérale de Lausanne (EPFL) Lausanne CH‐1015 Switzerland; ^3^ Vinca Institute of Nuclear Sciences University of Belgrade P.O.Box 522 Belgrade 11000 Serbia; ^4^ Department of Physics Indian Institute of Technology Delhi, Hauz Khas New Delhi 110016 India; ^5^ Department of Physics Indian Institute of Science Bangalore 560012 India; ^6^ CAS Key Laboratory of Magnetic Materials and Devices, Ningbo Institute of Materials Technology and Engineering Chinese Academy of Sciences Ningbo Zhejiang 315201 China; ^7^ Laboratory for Multiscale Materials Experiments Paul Scherrer Institut Villigen CH‐5232 Switzerland; ^8^ Paul Scherrer Institute Villigen CH‐5232 Switzerland

**Keywords:** magnetic coupling, metal–insulator transition, proximity effect

## Abstract

Employing X‐ray magnetic circular dichroism (XMCD), angle‐resolved photoemission spectroscopy (ARPES), and momentum‐resolved density fluctuation (MRDF) theory, the magnetic and electronic properties of ultrathin NdNiO_3_ (NNO) film in proximity to ferromagnetic (FM) La_0.67_Sr_0.33_MnO_3_ (LSMO) layer are investigated. The experimental data shows the direct magnetic coupling between the nickelate film and the manganite layer which causes an unusual ferromagnetic (FM) phase in NNO. Moreover, it is shown the metal–insulator transition in the NNO layer, identified by an abrupt suppression of ARPES spectral weight near the Fermi level (*E*_F_), is absent. This observation suggests that the insulating AFM ground state is quenched in proximity to the FM layer. Combining the experimental data (XMCD and AREPS) with the momentum‐resolved density fluctuation calculation (MRDF) reveals a direct link between the MIT and the magnetic orders in NNO systems. This work demonstrates that the proximity layer order can be broadly used to modify physical properties and enrich the phase diagram of RENiO_3_ (RE = rare‐earth element).

## Graphical abstract

The ferromagnetic order in NdNiO_3_ (NNO) film is induced by the stray field from the La_0.67_Sr_0.33_MnO_3_ (LSMO) layer in proximity. Consequently, the magnetic coupling between the NNO film and the LSMO layer melts the metal–insulator transition in the NNO layer. Thus, this study illuminates the intimate connection between magnetic order and electronic properties in RENO.

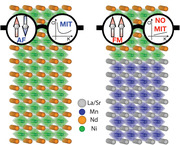

## 1 Introduction

**Figure 1:**
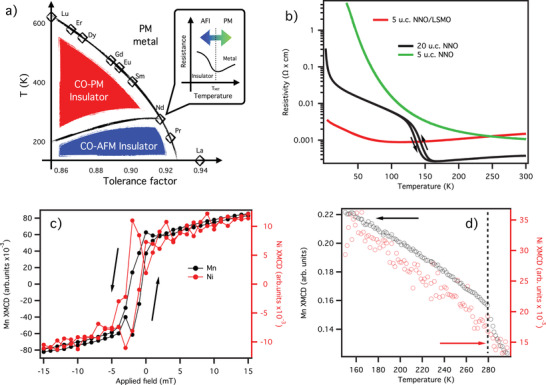
a) RENiO_3_ phase diagram. The inset qualitatively depicts how MIT is shifted via the proximity to the magnetic orderings. b) Temperature‐dependent resistivity data on NdNiO_3_ systems. The bulk‐like 20 u.c. NNO/NGO (black line) reference system undergoes a metal–insulator transition at 150 K. The second reference system, 5 u.c. NNO/NGO (green line), exhibits insulating behavior through the whole investigated temperature range. The 5 u.c. NNO/LSMO/NGO resistivity data show a softened MIT (red line). c) Magnetization loops measured by XMCD on the 5 u.c. NNO/LSMO/NGO sample on the Mn (black lines) and Ni (red lines) edges at 25 K. Their magnetization is in phase, indicating ferromagnetic coupling between the two layers. The magnetization curve is not centered around zero due to the flux trapped in the superconducting coils. d) Temperature dependence of Mn magnetization (black lines) and Ni (red lines) in the 5 u.c. NNO/LSMO/NGO sample under 6.5 T magnetic field: quenching of the FM ordering in LSMO above its *T*_C_ results in the same quenching in NNO.

The perovskite rare‐earth nickelates (RENiO_3_, RE = rare‐earth element) exhibit a wide variety of physical properties^[^ ^1^, ^2^, ^3^ ^]^ making them of great interest, not only from a theoretical standpoint, but also in terms of technological applications.^[^ ^4^, ^5^, ^6^, ^7^ ^]^ In these systems, the complex interplay between the spin, charge, and orbital degrees of freedom plays a crucial role in determining their physical properties and driving phase transitions.^[^ ^8^, ^9^, ^10^, ^11^ ^]^ The rare‐earth nickelates in their bulk form transition from a high‐*T* paramagnetic metal (PM‐M) phase to a paramagnetic insulator (PM‐I) phase below the metal–insulator transition (MIT) temperature, *T*_MI_. With further cooling, they become antiferromagnetic insulators (AF‐I) at the Néel temperature, *T*_N_. Reducing the size of the rare earth element brings *T*_MI_ and *T*_N_ closer, and for RE = Nd and Pr, *T*_MI_ and *T*_N_ merge (**Figure** **1**a), suggesting, in these cases, an intimate relation between magnetic ordering and electronic behavior. However, despite numerous experimental and theoretical studies, the origin of the MIT and the nature of the antiferromagnetic (AFM) insulating phase are still under debate.^[^ ^10^, ^12^, ^13^, ^14^, ^15^, ^16^, ^17^, ^18^, ^19^, ^20^ ^]^ One member of RENiO_3_ family, NdNiO_3_ (NNO), lies near the triple point of the *T* versus tolerance factor phase diagram,^[^ ^8^, ^9^ ^]^ where the direct PM‐M to AF‐I transition occurs (see Figure 1a). This property and the fact that the transition occurs at a very comfortable temperature for ordinary experiments (*T*_MI_ = *T*_N_ = 200 K), indeed grant NNO for studying an interplay and the relation between magnetism and the MIT.

Thin films and heterostructures of NNO offer new possibilities of altering the MIT via strain (compressive or tensile), film thickness^[^ ^7^, ^21^, ^22^, ^23^, ^24^, ^25^ ^]^ and/or interlayer charge transfer.^[^ ^26^ ^]^ However, in addition to noticeable MIT change, the AFM ordering in NNO might also be modified solely by reducing the film thickness, similarly to the effect observed in LNO based superlattices.^[^ ^27^ ^]^ Indeed, it was reported that both electronic and magnetic properties of NNO film in the proximity to a magnetic layer are strongly affected.^[^ ^26^, ^28^ ^]^ Moreover, various complex heterostructures created from NNO and other TMOs (CoFe_2_O_4_, LSMO, NdMnO_3_) showed the emergence of interfacial ferromagnetism.^[^ ^26^, ^29^, ^30^ ^]^ Therefore, it is crucial to disentangle the size effect from the interlayer charge transfer and potentially establish a link between the electronic structure (and MIT) and a magnetic ordering when the thin NNO film is magnetically perturbed.

In this study, we report the emergence of a ferromagnetic phase in NNO thin films in proximity with the ferromagnetic metal (FM‐M) La_0.67_Sr_0.33_MnO_3_ (LSMO) layer, as confirmed by X‐ray magnetic circular dichroism (XMCD) on the Ni edge. Besides, the same heterostructure shows a suppressed MIT, with a persisting density of states (DOS) at the Fermi level (FL) down to low temperature, as seen by angle‐resolved photoemission spectroscopy (ARPES). Finally, momentum‐resolved density fluctuation theory (MRDFT) allows us to establish the link between two phases, showing that the emergence of ferromagnetic ordering in NNO suppresses the opening of a AF gap in the DOS in its ground state.

## 2 Results

The focus of this work was on five unit cells (u.c.) NNO film grown on top of 15 u.c. LSMO (buffer) layer over NdGaO_3_ (NGO) (110) single crystal (in the following denoted as NNO/LSMO). All samples were grown by pulsed laser deposition (PLD): growth and transport details are in the ref. [21] and Supporting Information. Electronic and magnetic structures of this system were compared with those obtained from two NNO films directly grown over NGO (110) substrate. The first sample, 20 u.c. NNO film (thick‐NNO/NGO) mirrors the bulk properties, while 5 u.c. NNO film on NGO (thin‐NNO/NGO) was used to check a dimensionality (a size) effect on the electronic structure.

Our bulk‐like reference sample (black line in Figure 1b) shows a sharp MIT at 140 K, with a change of resistivity spanning more than one order of magnitude. In contrast, the second reference sample, thin‐NNO/NGO (green line in Figure 1b), displays a temperature‐independent insulating behavior, typical of many ultra‐thin oxide films. The NNO/LSMO sample shows a peculiar behavior instead with no clear sign of the MIT, while the resistivity changes less than a factor of two over the entire temperature range (4–300 K). The values of resistivity for 5 u.c. NNO/NGO and 5 u.c NNO/LSMO/NGO samples are comparable at higher *T* while at lower temperatures the first one exhibits a robust insulator behavior. However, the 5 u.c NNO/LSMO/NGO displays a rather soft sign of the MIT while remaining very conductive at low temperatures. Comparing the resistivity data of thick NNO film with 5 u.c NNO/LSMO sample at low T, it is clear that the former is much more metallic although both have a very comparable trend upon cooling down (excluding the part where MIT in thick NNO film occurs). However, we measured the total resistivity of the bilayer, and it is challenging to disentangle the contribution of NNO and LSMO layers. Overall, our transport data indicates that the FM underlayer alters the physical properties of the NNO layer.

To understand this response, we investigated the magnetic properties of our samples employing the XMCD method. In comparison with other magnetization probes, this experimental technique has the advantage of being element‐specific, allowing us to separate contributions to the magnetism between the two different layers (NNO and LSMO) in one sample, which cannot be achieved with classical transport methods.

Figure 1c shows the magnetization loops acquired at the Mn (black lines) and Ni (red lines) edges (see Supporting Information for experimental details). The observed hysteresis loops with the clear opening confirm ferromagnetism in LSMO layer, and unveil a novel ferromagnetic ordering in NNO. Moreover, the magnetic moments in the two layers are directly coupled, as evidenced by their temperature dependence reported in Figure 1d: across the 280 K *T*_C_ of LSMO magnetic moment is quenched, followed by the Ni one.

To investigate the effect of the emergence of this ferromagnetic order on the NNO electronic structure and, thus, MIT, we have employed ARPES method. The ARPES is the direct method to probe the *E* versus *k* electronic structure of solids, generating valuable information about the symmetries, topologies, and interactions.

**Figure 2:**
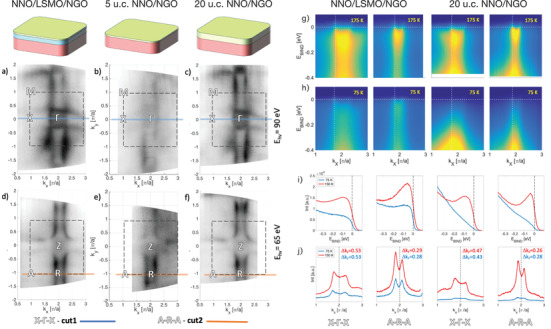
Fermi surfaces (FSs) at high temperature (*T* = 150 K). a–c) FSs measured at *hν* = 90 eV (*k*_*z*_ ≈ 0) and d–f) *hν* = 65 eV (*k*_*z*_ ≈ *π*) for NNO/LSMO, thin‐NNO/NGO, and thick‐NNO/NGO, respectively. Although the 5 u.c. NNO/NGO system is a strong insulator, we have been able to perform the ARPES measurement but only at high temperature. The photoemission intensity has been integrated over ±20 meV. The dashed square represents the zone boundary. The solid lines (blue on (d–f) and orange on (g–i)) represent the direction of high statistic cuts. g) Cut#1 along the X–Γ–X, and h) cut #2 along A–R–A directions from NNO/LSMO/NGO and thick‐NNO/NGO. Vertical dashed lines in (g,h) indicate the *k*_F_ positions where the EDC cuts were taken, shown in (i). Horizontal dashed lines in (g,h) indicate the position of the Fermi level and MDC cuts shown in (j).

In **Figure** **2** the Fermi surface (FS) cuts from the NNO/LSMO sample (panels a and d) is compared with the ones of the thin‐NNO/NGO (panels b and e) and thick‐NNO/NGO samples (panels c and f) acquired at 175 K (temperature above MIT). The FSs cuts in M–Γ–X plane are shown in panels a, b, c, while the FSs cuts in A–R–Z plane are displayed in d, e, and f. All three samples show the same FS topology, with cuboids centered at A point and a sphere at the Γ point, as expected.^[^ ^21^ ^]^ The direct comparison of these data shows that the electronic structure of the NNO layer in proximity to the LSMO layer does not significantly change at 175 K.

To further assess the nature of the FS and temperature evolution of the band structure, we examined the low‐energy band dispersions for all systems (Figure 2 panels g and h). While ARPES was possible to perform on the NNO/LSMO and the thick NNO/NGO samples at any temperatures, 5 u.c. NNO/NGO system suffered from severe charging problems at low temperatures (below 100 K). This consequence agrees with the transport data, displaying very high resistance in the thin NNO film at lower temperatures. The band dispersion maps were obtained by recording ARPES spectra parallel to two high‐symmetry lines: X–Γ–X (obtained at *hν* = 90 eV) and A–R–A (obtained at *hν* = 65 eV). The spectra recorded along high symmetry cuts we will refer as cut#1 and cut#2, respectively, as indicated in Figure 2. The 2D Fermi surfaces contures obtained from those spectra at the 150 K (for the thick NNO film temperature it is above MIT) verified that the small pocket at the Γ‐point exhibits electron‐like (convex parabolic‐like dispersion) behavior, while the large pocket at the BZ corner (*A* −point) is hole‐like (concave parabolic‐like dispersion). This observation is in agreement with previous observations^[^ ^21^ ^]^ of the nature of these bands (Figure 2 panels in rows g and h). A more comprehensive analysis of the low energy spectra at different temperatures is presented in Figure 2, panels i and j.

Both energy distribution curves (EDC) and momentum distribution curves (MDC) at high temperature (red line) and low temperature (blue line) for both samples are shown in Figure 2 panels in rows i and j. A well‐defined quasiparticle peak is visible in both distribution curves with substantial spectral weight at *E*_F_. The spectral weight near *E*_F_ in thick‐NNO/NGO is redistributed toward higher binding energy throughout the cooling process, indicating a gap opening below 75 K. Some of the co‐authors of this work already reported the comprehensive temperature dependence of EDCs and MDCs from 20 u.c. NNO grown on NGO (110).^[^ ^21^ ^]^ By contrast, the spectral weight near *E*_F_ in NNO/LSMO for cut#1 and cut#2 remains at low temperature forming a clear Fermi step. The NNO in proximity to the FM layer showed only the soft reduction of the spectral weight of quasiparticle bands down to 25 K (see Supporting Information for more whole MDCs and EDCs data). However, this soft reduction of the quasiparticle spectral weight suggests that considerable correlation effects emerge that can signify emerging magnetism and electron–phonon correlation. However, a softening of the spectral weight in the NNO/LSMO sample near the Fermi level during the cooling suggests that considerable correlation effects emerge. Indeed, the minima in transport data have been reported for metallic LNO thin films, where Scherwitzl and co‐workers attributed this behavior to weak localization.^[^ ^22^ ^]^ This effect might occur also in the NNO/LSMO sample, causing the observed upturning in the resistivity curve at a lower temperature.

It is worth noticing that the separation between the two peaks in the MDCs (denoted as Δ*k*_F_ panels in row j of Figure 2) is different in the two samples. In particular, the NNO/LSMO sample shows deviations in Δ*k*_F_ values than those in the thick‐NNO/NGO sample, indicating that the electron pocket increases while the hole pocket decreases. This observation concurs with an small upshift of the chemical potential and can be caused by electrons transfer from the LSMO to the NNO layer. The charge transfer across the interface was already announced to occur in NNO/TMO heterostructures. Saleem et al., reported ferromagnetic interaction between Ni–Ni and Ni–Co atoms in the NdNiO_3_/CoFe_2_O_4_ heterostructures caused by charge transfer from Co to Ni. Chen et al., reported that the Ni^3^^+^ cations (present in AF state) coexist with Ni^3^^+^ ions at the NNO/LSMO interface due to a charge transfer from the Mn cations. Consequently, the ferromagnetism at the interface emerges by the exchange coupling between Ni2^+^ and Mn4^+^ ions.^[^ ^26^ ^]^ Furthermore, it is also reported that interfacing the NNO layer with AF insulating (in its bulk form) NdMnO_3_ layer ferromagnetic interplays between Mn and Ni ions occurs at low T. Although the charge transfer cannot be foreseen directly, the authors speculate that an epitaxial strain in NdNiO_3_/NdMnO_3_ heterostructures is responsible for the different transport behavior. Dhaka et al., indeed found that a moderate compressive epitaxial strain very much alters the electronic structure and, therefore, transport properties on NNO film, primarily due to band reordering. Also, it is important to note that a bond disproportionation is the characteristic of RENO. Two varieties of octahedra, expended ‐ Ni 3d^8^ and collapsed ‐ Ni 3d^8^L^2^, are differently hybridized with the O2p hole states, resulting in an approximately equal amount of charges at the nickel sites.^[^ ^11^ ^]^

However, the causality between magnetic ordering in NNO and the MIT suppression remained elusively. To understand this ramification, we modeled the electronic structure of NNO with PM, AFM, and FM ordering using MRDF theory.^[^ ^31^, ^32^, ^33^ ^]^ This model captures the effects of symmetry breaking order parameters AFM and FM orders) on the metallic state in NNO.

Motivated by the XAS and XMCD results, we did not consider any other effect, such as a charge transfer or a direct exchange between magnetic moments in Mn and Ni ions, which might perturb the NNO layer in proximity to the LSMO.

We used a three‐band tight‐binding model for the non‐interacting low‐energy spectrum of NNO, which consists of $d_{x^{2}-y^{2}}$ and $d_{z^{2}}$ along with a hybridized t_2*g*_‐orbital with d_*xy*_ symmetry.^[^ ^21^ ^]^ See Supporting Information for a detailed description of the mean‐field model for the AFM and ferromagnetic states. Due to marginal density fluctuations, a strong self‐energy correction appears, leading to a band renormalization and a spectral weight distribution.^[^ ^31^, ^32^ ^]^

**Figure 3:**
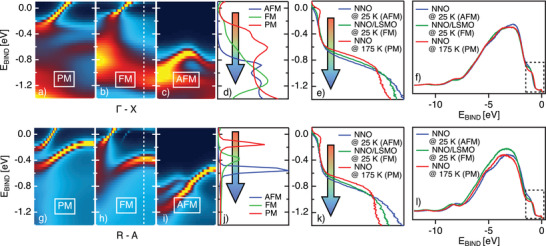
Calculated bend structure for NNO system in the a,g) PM, b,h) FM, and c,i) AFM phase along the Γ‐X and R‐A directions. d,j) Display the EDCs integrated at the zone boundary ((the range is indicated with the white box) of the calculated band structures for all three magnetic phases along with the Γ–X and R–A directions. e,k) The corresponding experimental spectra (EDCs) integrated at the zone boundary for the thick‐NNO sample at 175 K, and for NNO/LSMO and thick‐NNO sample at 20 K. The whole integrated VB at the zone boundary for all three samples are in (f, l). Blue boxes in those panels symbolize the zoom‐in shown in (e,k). Experimental spectra are directly comparable with the calculated and depict experimentally obtained EDCs of NNO in the PM, FM, and AFM phases.

The calculated spectra along the Γ–X (panels a, b, c) and R‐A (panel g, h, i) for paramagnetic (PM), ferromagnetic (FM), and AFM orderings are presented in **Figure** **3** (see Supporting Information for the spectral weight map along with all the high symmetry directions). Along both, the Γ–X and the R–A, directions the PM phase is characterized by two bands in the low energy region. The first one is with a strong quasiparticle attribute crosses the Fermi level (mainly with $d_{x^{2}-y^{2}}$ character), while the second less dispersing band is located at 0.5 eV of binding energy (primarily with $d_{z^{2}}$ character).

Our calculations are based on the interacting Hamiltonian consists of intra‐orbital (U), inter‐orbital (V), and Hund's coupling (*J*_H_), but neglects the weak pair‐hopping term. For simplicity, we consider all interaction parameters to be on‐site and orbital independent. In the AFM state, our model reproduces the “Mott–Hubbard” gap when the system is in AFM state (see Supporting Information), which is consistent with the insulator phase in this material. Nevertheless, introducing the FM ordering parameter, a fully insulating ground state does not emerge while the quasiparticle peak splits into upper and lower magnetic bands (U/LMBs). Consequently, the NNO‐FM at low *T* can be less metallic, accounting for spectral weight reduction from the region near the Fermi level seen by ARPES.

Although the quasiparticle band in the examined directions is barely affected by FM ordering, the $d_{z^{2}}$ band shifts to higher binding energy (BE) while AFM ordering causes the quasiparticle band folding accompanied by the gap opening, and the $d_{z^{2}}$ band shifts further down (1 eV BE). Panels d and j of the Figure 3 summarize these shifts for $d_{z^{2}}$ band through integrating EDCs for both cuts: the peak is shifting toward higher binding energy following a transition from the PM trough the FM to the AFM phases.

Our MRDF calculations do not depict O2p orbitals, and therefore a direct comparison with VB experimentally measured is challenging. Nevertheless, photoemission spectra indeed capture the trend indicated by theory, as shown in Figure 3 panels e and k. Here we compare integrated EDCs from the thick‐NNO measured experimentally along with the Γ–X and R–A directions at the high (above MIT), the low temperature (below MIT), and NNO/LSMO at low temperature. We assume that those data respectively represent PM, FM, and AFM phases. Along with both measured directions, the band located between 0.5 and 1 eV of binding energy shows the same trend of the shifting identified by the calculations. Following the evolution suggested by the MRDF calculations, we attribute this shift to a direct influence of the emerging magnetic orderings on the low energy spectrum of the NNO electronic structure. However, the experimentally observed electronic structure of the NNO layer in proximity to the FM‐LSMO agrees admirably with the calculated one utilized solely with the FM order parameter. Considering those mentioned above and the fact that a negligible charge transfer is observed, we argue that the novel FM magnetic order is induced initially by the stray field from the LSMO layer.

## 3 Conclusions

We studied the emergence of the ferromagnetic phase and its influence on the electronic structure in nickelate thin film in proximity to the magnetic layer. While temperature dependent XMCD data provided clear evidence of the direct ferromagnetic coupling between the nickelate film and the manganite layer, a combined MRDFT and ARPES method identified the signature in a band structure that is associated with the ferromagnetic phase in NNO. The interfacial ferromagnetism previously was explained as consequences of a charge transfer from FM layer to Ni sites.^[^ ^26^, ^30^ ^]^ However, our study shows that it can be induced in the NNO primarily by the stray field from the LSMO layer, which aligns the spins at Ni sites. We demonstrate that, in addition to strain and film thickness, the proximity layer emerges as an additional “knob” for manipulating existing and inducing novel physical properties, consequently ameliorating the phase diagram of *RE*NiO_3_.

## 4 Experimental Section

### Sample Preparation

The NNO thin films and NNO/LSMO bilayers were grown on NGO (110) single‐crystal substrates using a PLD method. A sintered stoichiometric NdNiO_3_ and La_0.67_Sr_0.33_MnO_3_ 2 inches pellets were used as an ablation target. The ablation was performed using the Nd:YAG (quadruple mode, 266 nm) with 90 mJ per pulse at a repetition rate of 2 Hz. The substrates were heated by a direct current passing through silicon wafers mounted beneath. The growth temperature was fixed to 730 °C (controlled by a pyrometer) while the oxygen pressure was 0.1 mbar during the deposition. After deposition, all samples were cooled down with a rate of 15° min^−1^ while maintaining the same oxygen pressure for about 1 h. The direct UHV connection between the PLD setup and the ARPES end‐station at the surface and interface spectroscopy (SIS) beamline (SLS) allowed in situ transfer of freshly grown sample and characterization of its electronic structure by ARPES.^[^ ^34^ ^]^

### Angle‐Resolved Photoemission

All the ARPES data presented were measured at the high‐resolution angle‐resolved photoemission spectroscopy endstation at SIS beamline of the Swiss Light Source. The photoelectron analyzer in use was a Scienta R4000 hemispherical analyzer. The sample was cooled by a liquid helium cryostat which allowed measurements at temperatures as low as 15 K, while higher temperatures were achieved by slowly heating the cryostat away from the sample. This method allowed changing the temperature without significantly influencing the pressure.

### X‐Ray Magnetic Circular Dichroism

The XMCD ex situ measurements at EPFL/PSI X‐Treme beamline^[^ ^35^ ^]^ had been performed on freshly grown NNO films and NNO/LSMO bilayers. The end‐station was equipped with a split‐pair of superconducting coils to apply up to 7 T along the X‐ray beam. The variable temperature was based on pumped He‐4 cryostat, allowing it to reach 2–3 K at the sample. The measurements had been performed in total electron yield mode with a resolution of around 0.1 eV. Absorption spectra at fixed magnetic field values were acquired alternating light with right‐handed and left‐handed circular polarization.

### Momentum‐Resolved Density Fluctuation Calculation

The electronic structure of NNO was modeled with PM, AFM, and FM ordering using MRDF theory. Three orbitals, $d_{x^{2}-y^{2}}$, $d_{z^{2}}$, and d_*xy*_ had been used for the tight‐binding model (for details, see Supporting Information).

## Conflict of Interest

The authors declare no conflict of interest.

## Supporting information

Supporting Information

## Data Availability

Research data are not shared.
