# Supplementary material for: Proximity‐Induced Novel Ferromagnetism Accompanied with Resolute Metallicity in NdNiO3 Heterostructure
Source: Adv Sci (Weinh). 2021 Aug 11;8(19):2101516. doi: 10.1002/advs.202101516 (PMC8498901; doi:10.1002/advs.202101516)
Supplement: Supplementary file 1 — Supporting Information [file ADVS-8-2101516-s001.pdf]

SUPPLEMENTARY INFORMATION

Proximity-induced novel ferromagnetism accompanied with resolute metallicity in $NdNiO_3$ heterostructure

M. Caputo,[1,2,*] Z. Ristic,[1,2,3,*] R. S. Dhaka,[1,2,4] Tanmoy Das,[5] Z. Wang,[1,6] C. E. Matt,[1] N. C. Plumb,[1] E. Bonini Guedes,[1,2] J. Jandke,[1] M. Naamneh,[1] A. Zakharova,[1] M. Medarde,[7] M. Shi,[1] L. Patthey,[1] J. Mesot,[8,2] C. Piamonteze,[1] and M. Radovic[1,†]

[1]*Photon Science Department, Paul Scherrer Institute, CH-5232 Villigen PSI, Switzerland*
[2]*Institute of Condensed Matter Physics, Ecole Polytechnique Fédérale de Lausanne (EPFL), CH-1015 Lausanne, Switzerland*
[3]*Laboratory for Radiation Chemistry and Physics, VINCA Institute of Nuclear Sciences, Belgrade University, 11001 Belgrade, Serbia*
[4]*Department of Physics, Indian Institute of Technology Delhi, Hauz Khas, New Delhi-110016, India*
[5]*Department of Physics, Indian Institute of Science, Bangalore 560012, India*
[6]*CAS Key Laboratory of Magnetic Materials and Devices, Ningbo Institute of Materials Technology and Engineering, Chinese Academy of Sciences, Ningbo, Zhejiang 315201, China*
[7]*Laboratory for Multiscale Materials Experiments, Paul Scherrer Institut, CH-5232 Villigen PSI, Switzerland*
[8]*Paul Scherrer Institute, CH-5232 Villigen PSI, Switzerland*
(Dated: May 28, 2021)

SAMPLE GROWTH AND CHARACTERISATIONS

The study has been performed at the Surface/Interface Spectroscopy (SIS) X09LA and X-Treme X07MA beamlines of the Swiss Light Source located at the Paul Scherrer Institute in Villigen, Switzerland. The PLD+ARPES facility at the SIS beamline provides such conditions that thin films grown by pulsed laser deposition (PLD) can be transferred to the ARPES endstation without breaking ultrahigh vacuum conditions, while films were transferred *ex-situ* to the X-Treme experimental chamber for XAS and XMCD measurements. The film thicknesses and surface ordering are routinely monitored with single-layer accuracy by reflection high-energy electron diffraction (RHEED).

We have investigated 5 and 20 unit cells (u.c.) of NNO (thin and thick NNO respectively) on $NdGaO_3$ substrates and in proximity with 15 u.c. of ferromagnetic metallic LSMO (NNO/LSMO). The growth parameters for LSMO were identical to those used for growing NNO films Ref. [1]: T_d = 720 C, $P(O_2)$= 0.1 mbar.

Epitaxial strain can alter band structures and electronic and magnetic properties of NNO film. We have investigated the strain effect on the electronic structure in detail in our previous work [1]. There we found that the electronic structure of the NNO film under the tensile strain of + 1.4% (NGO 110 substrate) is not much different than for the NNO bulk. Nevertheless, the compressive strain of only - 0.3% (LAO 001 substrate) has a much more dramatic effect for the NNO film. It causes a charge redistribution between electrons and holes (the hole pocket of FS centred at A points expanded), and "pushes" one band (before fully occupied) to cross Ef at A point. This affects the electronic properties of the NNO/LAO system, suppressing the MIT dramatically. Knowing that the NNO/NGO (110) heterostructure pictures well the bulk properties of the NNO, our work presented in the manuscript under review was focused on this model system.

Figs. 1 shows the time evolution of RHEED specular spot intensity (panels a–c) during a growth and RHEED end-patterns (panels d–f). In all the thress systems, RHEED pattern indicates that our surface is atomically flat and with orthorhombic order. The RHEED oscillation allows us to determine the thickness of our NNO and LSMO films.

Temperature-dependent resistivity data on the LSMO/NGO and NNO/LSMO/NGO are presented in the left panel of Fig. 2. In order to maximise the NNO transport contribution, we deposited four gold contacts on the top of NNO layers and attached Al wires on the contacts using a wire bonder (See inset in Figure 2).

ARPES MEASUREMENTS

The freshly grown samples were studied *in-situ* using the ARPES endstation at the Surface/Interface Spectroscopy (SIS) X09LA beamline. The endstation is equipped with a VG-Scienta R4000 electron energy analyzer and a six axis liquid He-cooled manipulator (CARVING). We used photon energies from 60 eV to 115 eV with both circular

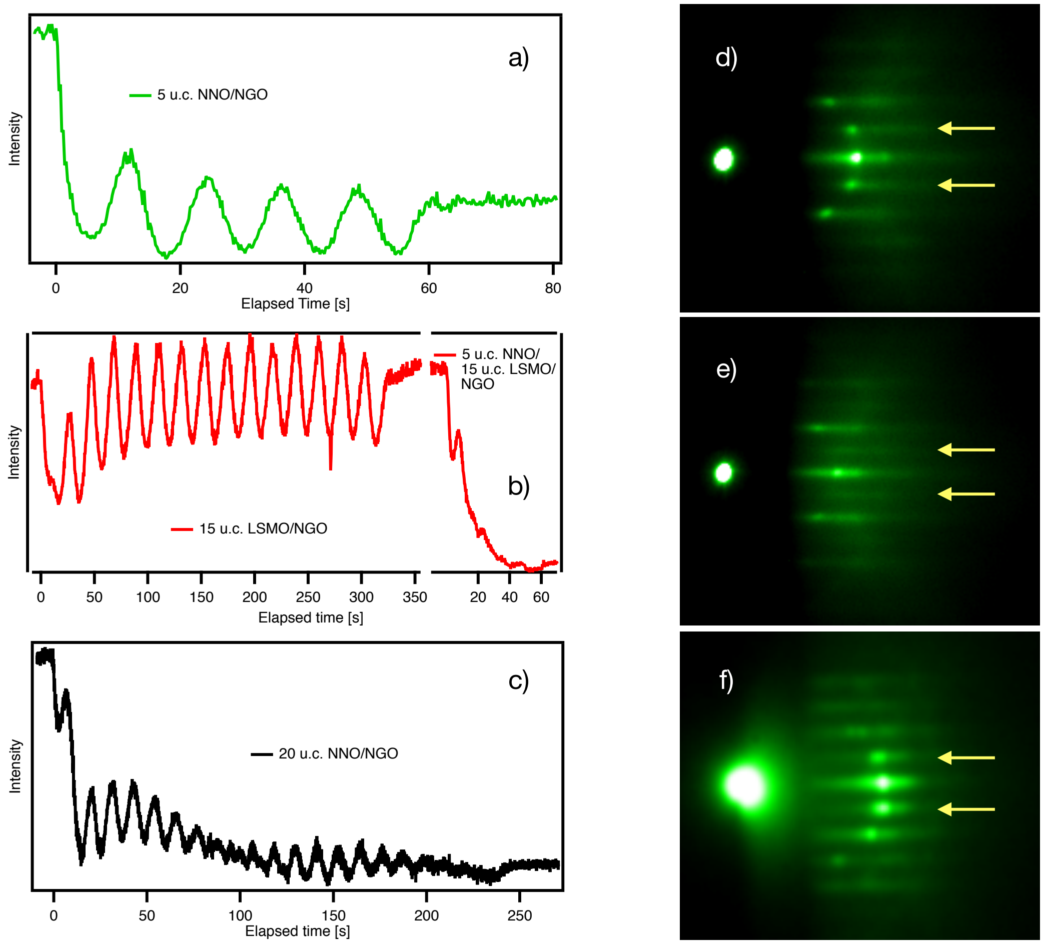

FIG. 1. RHEED patterns acquired at the end of the growth (panels d–f) and reflected spot intensity oscillations of the studied films (panels a–c). Yellow arrows in panels d–f point toward the 2x1 reconstruction observed in the RHEED pattern: this is a signature of the orthorhombic unit cell of NNO

and linear photon polarizations. Spectra were acquired over a temperature range from 20 to 200 K. The energy and momentum resolutions were set to about 20 meV and $\approx 0.009/0.019$ Å^{-1} (parallel/perpendicular to the analyzer slit), respectively. The binding energy scale was calibrated with a polycrystalline copper reference sample in direct electrical and thermal contact with the film. The base pressure of the UHV chamber during the measurements was below $5{\times}10^{-11}$ mbar.

Despite the ultra-thin limit of the top NNO layer, it is worth pointing out that the electron inelastic mean free path of electrons in the energy range used in this study is in the order of few Å[6], therefore much smaller than the NNO thickness. However, both 5 u.c. NNO layers show very similar FS topology with thick NNO film, formed by cuboids centered at A points and a sphere centered at the Γ point, showing 3D character of the band structure. Indeed, the band structures near EF for all 5 u.c. NNO layers grown on LSMO or just NGO are practically identical to the band structure of thick NNO film [1]. Based on these factors, we are very confident that the measured spectral weight in our ARPES data represents the whole NNO layer, not just the surface.

DETAILED TEMPERATURE DEPENDENT NNO/LSMO QUASIPARTICLE EVOLUTION

The nature of the electronic structure and its temperature evolution were depicted through the low-energy band dispersions acquired at 8 different temperatures for the 5 u.c. NNO/LSMO/NGO. Here we discuss only EDCs and MDc presented in Figure 3. The NNO in proximity to FM layer showed only the soft reduction of the spectral weight of quasiparticle bands down to 25 K. However, this soft reduction of the spectral weight can be a sing of emerging

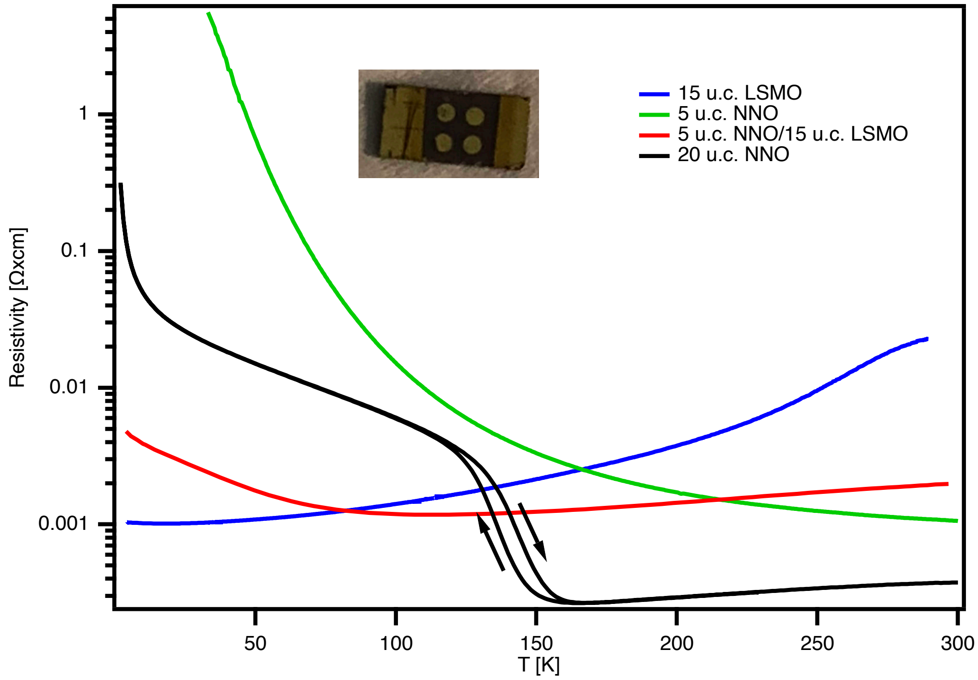

FIG. 2. Resistivity-vs.-temperature data for the studied films.

magnetism and/or electron - phonon correlation.

In the AFM state, our model reproduces the "Mott-Hubbard" gap when the system is in AFM state (See Figure 7), which is consistent with the insulator phase in this material. By contrast, for any value of the FM ordering parameter, a fully insulating ground state does not emerge while the quasiparticle peak splits into upper and lower magnetic bands (U/LMBs). Therefore, the NNO-FM at low T can be less metallic, which can account for the spectral weight reduction from the region near the Fermi level seen by ARPES. In addition, the reduced spectral weight is observed in layered (2D) LSMO system in the metallic state, due to electron-phonon interaction [8]

XMCD MEASUREMENTS

Freshly grown $NdNiO_3$ based heterostructures where transferred *ex-situ* for XMCD measurements in the EPFL/PSI X-Treme beamline [2]. The end-station is equipped with a split-pair of superconducting coils to apply up to 7T along the x-ray beam. The variable temperature insert consists of a pumped He-4 cryostat, allowing it to reach 2-3K at the sample.

Figure 4 shows typical XMCD spectra at Ni and Mn L3,2 edges for the NNO/LSMO bilayer. The intense peak at ca. 849eV corresponds to La M4 edge. The measurements have been performed in total electron yield mode with the resolution around 0.1eV.

Absorption spectra at fixed magnetic field values were acquired alternating light with right-handed and left-handed circular polarisation (C^+ and C^- respectively). XAS spectra (top panels) are then calculated simply $XAS = C^+ + C^-$, while $XMCD = C^+ - C^-$. In figure 1d of the main text the XMCD around the L3 edge was measured in short scans while slowly ramping the temperature. All measurements were performed after zero field cooling.

The magnetisation curves shown in figure 1c were measured by detecting the total electron yield as a function of applied field at two energies: at the maximum XMCD contrast and at the pre-edge where no XMCD contrast is found. The measurement is repeated for both x-ray helicity and both sweep field directions. The shift of the magnetisation curve in the applied field axis is caused by the flux trapped in the superconducting coils.

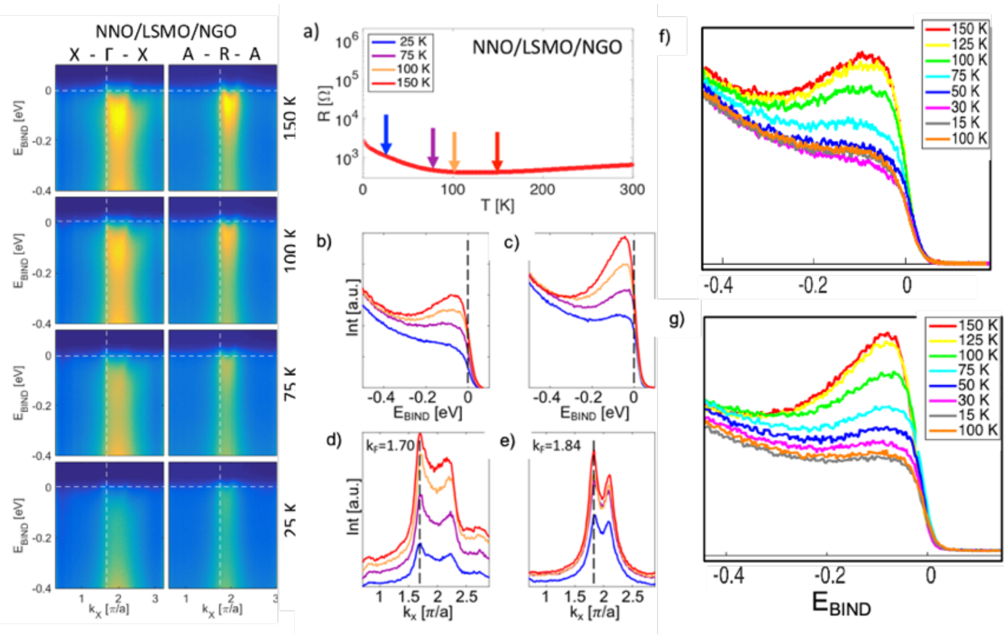

FIG. 3. Transport measurements of NNO/LSMO sample with indicated temperatures (color coded arrows) at which ARPES measurements were performed. Left: ARPES spectra. (b–e) EDC and MDC cuts obtained at different temperatures.

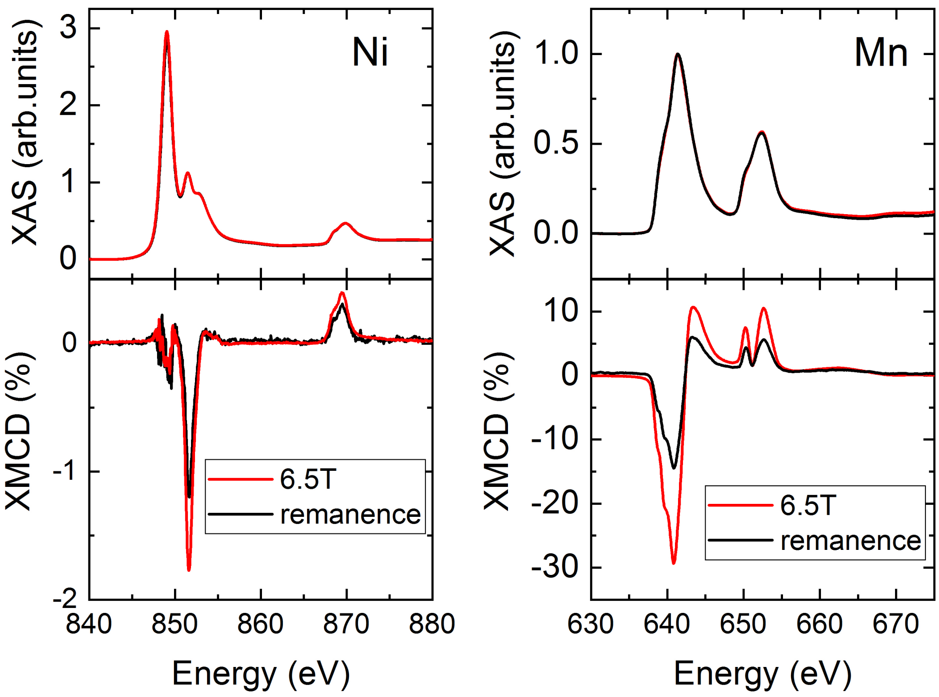

FIG. 4. XAS and XMCD measurements examples on the Ni and Mn edge acquired on the NNO/LSMO sample.

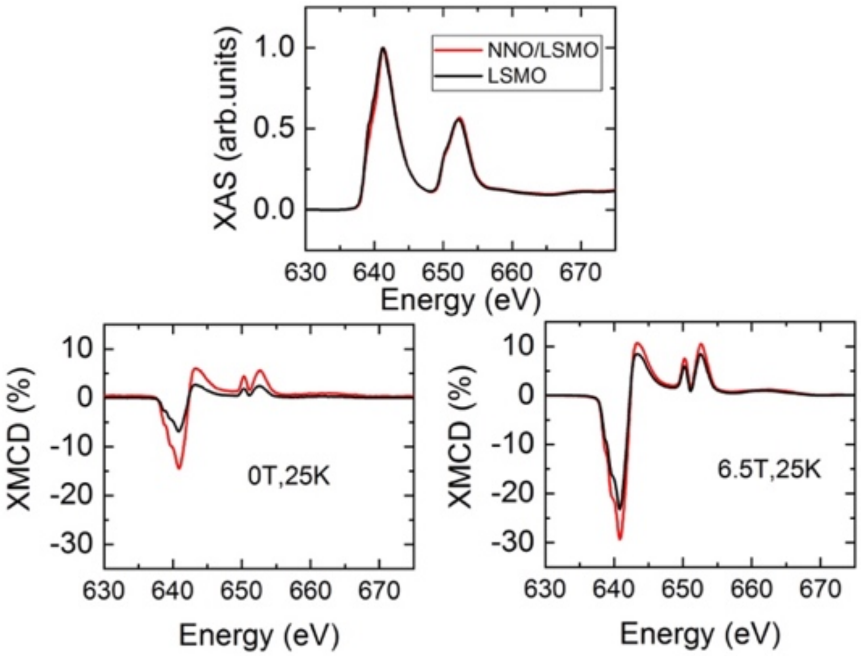

FIG. 5. Top panel: XAS at Mn L3,2 edges for NNO/LSMO bilayer and LSMO single film. In both cases LSMO is 15 u.c. thick and deposited on NGO (110). Bottom, left: XMCD in remanence, after applying 6.5T. Bottom, right: XMCD at 6.5T applied field. All measurements are at 25K.

We have characterized both NNO and LSMO single films grown on NGO (110) by XAS and XMCD. The measurements are shown in the figures below.

Figure 5 displays XAS and XMCD of Mn in the bilayer versus single LSMO layer. The probed magnetism in LSMO is improved by the capping with the nickelate as already observed in similar nickelate/manganite heterostructures [9]. Other than that, both systems are very similar in the shape of the XAS and XMCD.

Figure 6 shows the XAS and XMCD measured at the Ni edge for NNO/LSMO compared to NNO single layer. An XMCD signal is observed in NNO single layer when magnetic field is applied. This magnetization is however not remanent, as shown in figure 2c. The existence of a magnetic signal with applied field which turns out to be non-remanent indicates there could be some paramagnetic moments in Ni. Those paramagnetic moments could come from the top surface or interface with LSMO. This figure exemplifies well how different the magnetic behavior of single layer and bilayer are.

MEAN-FIELD MODEL FOR THE ANTIFERROMAGNETIC AND FERROMAGNETIC STATES

We use a three band tight-binding model for the non-interacting low-energy spectrum of NNO, which consists of $d_{x^2-y^2}$ and d_{z^2} orbitals, along with a hybridized t_{2g}-orbital with d_{xy} symmetry [1].The corresponding Hamiltonian, H_0 is

$$H_0 = \sum_{\mathbf{k},\sigma} \xi_i(\mathbf{k}) c^{\dagger}_{i,\mathbf{k}\sigma} c^{\dagger}_{i,\mathbf{k}\sigma} + \xi_{ij}(\mathbf{k}) c^{\dagger}_{i,\mathbf{k}\sigma} c^{\dagger}_{j,\mathbf{k}\sigma}, \tag{1}$$

where the first and second terms are the intra- and the inter-orbital hopping terms, respectively. $c^{\dagger}_{i,\mathbf{k}\sigma}$ ($c_{i,\mathbf{k}\sigma}$) is the creation (annihilation) operator for an orbital i at crystal momentum $\mathbf{k}_1$ with spin $\sigma =\uparrow$ or $\downarrow$, where $\bar{\sigma}$ corresponds

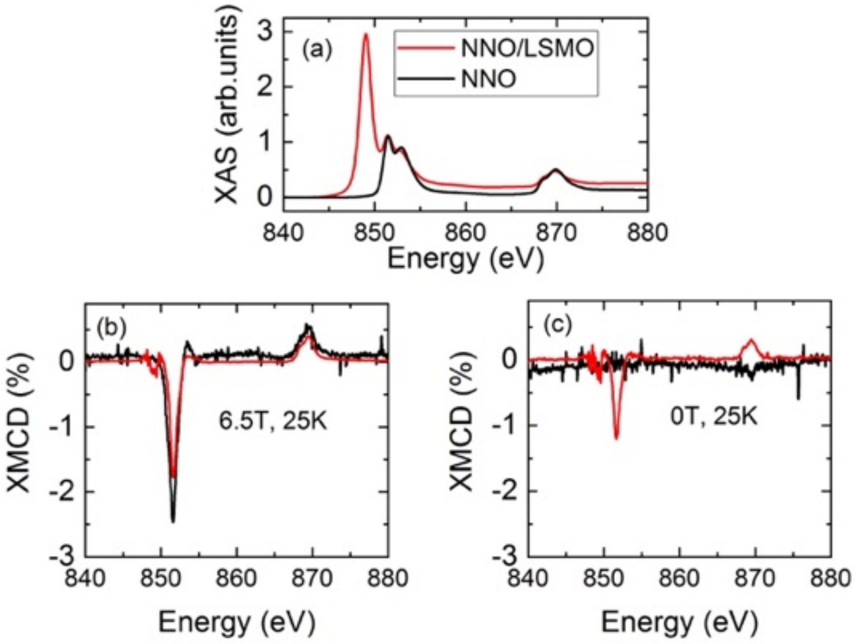

FIG. 6. Top row: XAS at Ni L3,2 edges for NNO/LSMO and NNO (20 u.c.), deposited on NGO (110). Bottom row, left: XMCD measured at applied field of 6.5T. Bottom, right: XMCD measured in remanence, after applying magnetic field. All measurements at 25K.

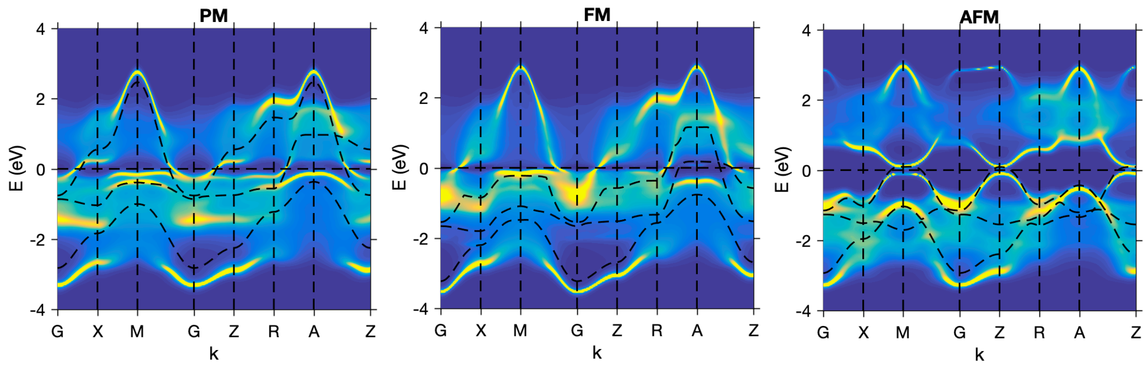

FIG. 7. Calculated spectral weight map including corresponding complex self-energy is plotted in blue (low) to red (high) colormap for paramagnetic (PM), antiferromagnetic (AFM), ferromagnetic (FM) phases. Black dashed lines give corresponding dispersions without the self-energy correction, but with the AFM and FM gap when applicable.

to opposite spin of σ. The non-interacting dispersions are [1]

$$
\begin{aligned}
\xi_1(\mathbf{k}) &= -2t_1(\cos k_x + \cos k_y) - 4t_2 \cos k_x \cos k_y + \delta_1 - \mu, \\
\xi_2(\mathbf{k}) &= -2t_3(\cos k_x + \cos k_y) - 4t_4 \cos k_x \cos k_y - 2t_5 \cos k_z + \delta_2 - \mu, \\
\xi_3(\mathbf{k}) &= -2t_6(\cos k_x + \cos k_y) - 2t_7 \cos k_z + \delta_3 - \mu,
\end{aligned} \tag{2}
$$

and

$$\begin{aligned}\xi_{12}(\mathbf{k}) &= -2t_8(\cos k_x - \cos k_y),\\ \xi_{13}(\mathbf{k}) &= -2t_9(\cos k_x + \cos k_y),\\ \xi_{23}(\mathbf{k}) &= -2t_{10}\cos k_x \cos k_y.\end{aligned} \tag{3}$$

t_i are various nearest and next-nearest neighbor hoppings, δ_i are the onsite potentials for different orbitals, and μ is the system's chemical potential. The corresponding tight-binding parameters are obtained by fitting the eigenvalues of the above Hamiltonian to the corresponding DFT band structure[1]. Hence we obtain t_1=0.41, t_2=-0.12, δ_1=0, t_3=0.05, t_4=0.04, t_5=0.35, δ_2=-0.1, t_6=0.23, t_7=0.15, δ_3=-1.8, t_8=0.2, t_9=0.1, t_{10}=0.1, all in eV [1].

The interaction effect has two parts. First, due to nesting and proximity effects, magnetic order parameter(s) develops in the low-energy spectrum, which is treated within Hartree-Fock formalism. Again due to magnetic fluctuations, the momentum-dependent self-energy correction gives band renormalization and spectral weight distribution which is computed within the momentum-resolved density fluctuation (MRDF) theorem.[3] For the multiband structure, the interacting Hamiltonian consists of intra-orbital (U), inter-orbital (V) and Hund's coupling (J_H) interactions, but neglects the weak pair-hopping term. For simplicity, we consider all interaction parameters to be onsite and orbital independent. So we obtain,

$$H_{\rm int} = U\sum_i n_{i\uparrow}n_{i\downarrow} + V\sum_{i\neq j} n_i n_j - J_H\sum_{i\neq j}\mathbf{S}_i\cdot\mathbf{S}_j, \tag{4}$$

where i and j are orbital indices. Taking $n_i = n_{i\uparrow} + n_{i\downarrow}$, and fixing the spin orientation axis along the $z-$direction, we have $S_{iz} = n_{i\uparrow} - n_{i\downarrow}$. Now replacing the number operator with $n_{i\sigma} = \sum_{\mathbf{k}} c^{\dagger}_{i,\mathbf{k}\sigma}c^{\dagger}_{i,\mathbf{k}\sigma}$, arrive at

$$\begin{aligned}H_{\rm int} &= U\sum_i n_{i\uparrow}n_{i\downarrow} + (V-J_H)\sum_{i\neq j}(n_{i\uparrow}n_{j\uparrow} + n_{i\downarrow}n_{j\downarrow}) + (V+J_H)\sum_{i\neq j}(n_{i\uparrow}n_{j\downarrow} + n_{i\downarrow}n_{j\uparrow}),\\ &= \sum_{\mathbf{k},\mathbf{k}'}\Bigg[U\sum_i c^{\dagger}_{i,\mathbf{k}\uparrow}c_{i,\mathbf{k}\uparrow}c^{\dagger}_{i,\mathbf{k}'\downarrow}c_{i,\mathbf{k}'\downarrow} + (V-J_H)\sum_{i\neq j}\left(c^{\dagger}_{i,\mathbf{k}\uparrow}c_{i,\mathbf{k}\uparrow}c^{\dagger}_{j,\mathbf{k}'\uparrow}c_{j,\mathbf{k}'\uparrow} + \text{h.c.}\right)\\ &\quad +(V+J_H)\sum_{i\neq j}\left(c^{\dagger}_{i,\mathbf{k}\uparrow}c_{i,\mathbf{k}\uparrow}c^{\dagger}_{j,\mathbf{k}'\downarrow}c_{j,\mathbf{k}'\downarrow} + \text{h.c.}\right)\Bigg].\end{aligned} \tag{5}$$

For the antiferromagnetic (AFM) state, we know that the nesting vector is $\mathbf{Q}\sim(0.5, 0.5, 0.5)2\pi$, which arises from the nesting between the $d_{x^2-y^2}$ and d_{z^2} orbitals dominating the electron-pocket at the Γ-point, and the hole-pockets at the R-point. This result is consistent with the susceptibility calculation and with the previous ARPES experiment [1]. Based on these results, we introduce the AFM and FM order parameters as

$$\mathcal{O}_{\rm AFM} = \sum_{i\neq j,\mathbf{k}\sigma}\sigma\left\langle c^{\dagger}_{i,\mathbf{k},\sigma}c_{j,\mathbf{k}+\mathbf{Q},\sigma}\right\rangle, \tag{6}$$

$$\mathcal{O}_{\rm FM} = \sum_{i,\mathbf{k}\sigma}\sigma\left\langle c^{\dagger}_{i,\mathbf{k},\sigma}c_{i,\mathbf{k},\sigma}\right\rangle. \tag{7}$$

Employing the mean-field theory, we obtain the interaction Hamiltonians for the two cases as,

$$H^{\rm AFM}_{\rm int} = -(V-J_{\rm H})\mathcal{O}_{\rm AFM}\sum_{i,\mathbf{k}}\sigma c^{\dagger}_{i,\mathbf{k}\sigma}c_{i,\mathbf{k}+\mathbf{Q}\sigma} + (V-J_{\rm H})\mathcal{O}^2_{\rm AFM}, \tag{8}$$

$$H^{\rm FM}_{\rm int} = \mathcal{O}_{\rm FM}\sum_{i,j\neq i,\mathbf{k}}\left[U\sigma c^{\dagger}_{i,\mathbf{k}\sigma}c_{i,\mathbf{k}\sigma} + (V-J_{\rm H})\sigma c^{\dagger}_{j,\mathbf{k}\sigma}c_{j,\mathbf{k}\sigma} + (V+J_{\rm H})\sigma c^{\dagger}_{j,\mathbf{k}\bar{\sigma}}c_{j,\mathbf{k}\bar{\sigma}}\right] + (U+2V)\mathcal{O}^2_{\rm FM}, \tag{9}$$

where the spin index $\sigma = -\bar{\sigma} = \pm$. The corresponding quasiparticle gaps are defined as $\Delta_{\rm AFM} = (V-J_{\rm H})\mathcal{O}_{\rm AFM}$, and $\Delta_{\rm FM} = (U+2V)\mathcal{O}_{\rm FM}$, assuming the gap remains the same for both orbitals. For the AFM state we choose a Nambu' spinor as $\Psi^{\rm AFM}_{\mathbf{k}} = (c_{1,\mathbf{k}\uparrow}, c_{2,\mathbf{k}\uparrow}, c_{3,\mathbf{k}\uparrow}, c_{1,\mathbf{k}+\mathbf{Q}\uparrow}, c_{2,\mathbf{k}+\mathbf{Q}\uparrow}, c_{3,\mathbf{k}+\mathbf{Q}\uparrow})^{\dagger}$, and one for the FM state is $\Psi^{\rm FM}_{\mathbf{k}} =$

$(c_{1,\mathbf{k}\uparrow}, c_{2,\mathbf{k}\uparrow}, c_{3,\mathbf{k}\uparrow}, c_{1,\mathbf{k+Q}\uparrow}, c_{2,\mathbf{k+Q}\uparrow}, c_{3,\mathbf{k+Q}\uparrow})^{\dagger}$. In these basis, the corresponding total Hamiltonians read:

$$H^{\rm AFM}_{\rm tot}(\mathbf{k}) = \begin{pmatrix} \xi_1(\mathbf{k}) & \xi_{12}(\mathbf{k}) & \xi_{13}(\mathbf{k}) & 0 & \Delta_{\rm AFM} & 0 \\ \xi^*_{12}(\mathbf{k}) & \xi_2(\mathbf{k}) & \xi_{23}(\mathbf{k}) & \Delta_{\rm AFM} & 0 & 0 \\ \xi^*_{13}(\mathbf{k}) & \xi_{23}(\mathbf{k}) & \xi_3(\mathbf{k}) & 0 & 0 & 0 \\ 0 & \Delta_{\rm AFM} & 0 & \xi_1(\mathbf{k+Q}) & \xi_{12}(\mathbf{k+Q}) & \xi_{13}(\mathbf{k+Q}) \\ \Delta_{\rm AFM} & 0 & 0 & \xi^*_{12}(\mathbf{k+Q}) & \xi_2(\mathbf{k+Q}) & \xi_{23}(\mathbf{k+Q}) \\ 0 & 0 & 0 & \xi^*_{13}(\mathbf{k+Q}) & \xi_{23}(\mathbf{k+Q}) & \xi_3(\mathbf{k+Q}) \end{pmatrix}, \quad (10)$$

and for the FM, we have

$$H^{\rm FM}_{\rm tot}(\mathbf{k}) = \begin{pmatrix} \xi_1(\mathbf{k})+\Delta_{\rm FM} & \xi_{12}(\mathbf{k}) & \xi_{13}(\mathbf{k}) & 0 & 0 & 0 \\ \xi^*_{12}(\mathbf{k}) & \xi_2(\mathbf{k})+\Delta_{\rm FM} & \xi_{23}(\mathbf{k}) & 0 & 0 & 0 \\ \xi^*_{13}(\mathbf{k}) & \xi_{23}(\mathbf{k}) & \xi_3(\mathbf{k}) & 0 & 0 & 0 \\ 0 & 0 & 0 & \xi_1(\mathbf{k})-\Delta_{\rm FM} & \xi_{12}(\mathbf{k}) & \xi_{13}(\mathbf{k}) \\ 0 & 0 & 0 & \xi^*_{12}(\mathbf{k}) & \xi_2(\mathbf{k})-\Delta_{\rm FM} & \xi_{23}(\mathbf{k}) \\ 0 & 0 & 0 & \xi^*_{13}(\mathbf{k}) & \xi_{23}(\mathbf{k}) & \xi_3(\mathbf{k}) \end{pmatrix}. \quad (11)$$

Since the third orbital (hybridized d_{xy} orbital) does not cross the Fermi level in this system, it remains unaffected by the AFM FS nesting. For the same reason, we also do not consider the FM order for this orbital. An insulating gap occurs only in the AFM state for a critical value of $\Delta_{\rm AFM} \gtrsim 0.9$ eV, giving a quasiparticle gap in the band structure $\sim$50-60 meV, as also seen experimentally. In contrast, for any value of the FM gap, a fully insulating ground state does not occur in this case.

When the dynamical correlations due to spin-, charge-, and orbital density fluctuations are included, as discussed below, both AFM and FM bands split further by the corresponding self-energy correction. Such a four-band structure (for each orbital) is evident in the calculated spectrum. In the magnetic state, the magnetically split bands are referred to upper and lower magnetic bands (U/LMBs). The self-energy split bands are referred to here as upper and lower-Hubbard bands (U/LHBs). In the AFM state, our model reproduces the coexistence of the AFM and 'Mott-Hubbard' gap, in consistent with the two metal-insulator transitions in this material. In the FM state, the 'Mott-Hubbard' gap localizes the quasiparticle states by reducing their spectral weight from the low-energy region. Therefore, in the FM state, although a fully insulating quasiparticle gap does not open the insulating-like behavior arises due to spectral weight distributions.

ADDITIONAL CORRELATIONS TO THE MEAN-FIELD GROUND STATE

In an earlier study, we have demonstrated that the self-energy correction due to various density-density fluctuations leads to substantial renormalization of both quasiparticle bands and associated their spectral weights as a function of both energy and momentum in the paramagnetic state [1] Here we extend the momentum-resolved density fluctuation (MRDF) calculations[3–5] to various magnetic ground states discussed above.

The single-particle Green's function is defined as $\tilde{G}_0(\mathbf{k}, i\omega_{\mathfrak{n}}) = \left(i\omega_{\mathfrak{n}}\tilde{1} - \tilde{H}_{\rm tot}\right)^{-1}$, where $i\omega_{\mathfrak{n}}$ is the Matsubara frequency for the fermions, and $H_{\rm tot}$ is the total magnetic Hamiltonian defined in Eqs. (10, 11). The explicit form of G is then obtained as

$$G_{mn}(\mathbf{k}, i\omega_{\mathfrak{n}}) = \sum_{\nu} \frac{\phi^{\nu}_{\mathbf{k},m}\phi^{\nu\dagger}_{\mathbf{k},n}}{i\omega_{\mathfrak{n}} - E^{\nu}_{\mathbf{k}}}. \quad (12)$$

Here $\mathbf{q}$ and ω_p are the bosonic excitation momentum and frequency, respectively. $\phi^{\nu}_{\mathbf{k},m}$ is the eigenstate for the $\nu^{\rm th}$ tight-binding band ($E^{\nu}_{\mathbf{k}}$), projected onto the $m^{\rm th}$ orbital. The non-interacting density fluctuation susceptibility is

$$\chi^{st}_{0,mn}(\mathbf{q}, \omega_{\mathfrak{p}}) = -\frac{1}{\Omega_{\rm BZ}\beta} \sum_{\mathbf{k},\mathfrak{n}} G_{mn}(\mathbf{k}, i\omega_{\mathfrak{n}}) G_{st}(\mathbf{k+q}, i\omega_{\mathfrak{n}} + \omega_{\mathfrak{p}}), \quad (13)$$

where $\beta = 1/k_BT$, and k_B is the Boltzmann constant and T is temperature. $\Omega_{\rm BZ}$ is the electronic phase space volume. $f^{\nu}_{\mathbf{k}}$ and n_p are the fermion and boson occupation numbers, respectively. After performing the Matsubara summation over the fermionic frequency ω_n and taking analytical continuation to the real frequency as $\omega_{\mathfrak{n}} \rightarrow \omega + i\delta$, we get

$$\chi^{st}_{0,mn}(\mathbf{q}, \omega_{\mathfrak{p}}) = -\frac{1}{\Omega_{\rm BZ}} \sum_{\mathbf{k},\nu,\nu'} \phi^{\nu\dagger}_{\mathbf{k+q},s}\phi^{\nu}_{\mathbf{k+q},t}\phi^{\nu'}_{\mathbf{k},n}\phi^{\nu'\dagger}_{\mathbf{k},m} \frac{f^{\nu}_{\mathbf{k+q}} - f^{\nu'}_{\mathbf{k}}}{\omega_{\mathfrak{p}} + i\delta - E^{\nu'}_{\mathbf{k}} + E^{\nu}_{\mathbf{k+q}}}. \quad (14)$$

The RPA Hamiltonian for the multiband system is the same as the interacting Hamiltonian we have written above in Eq. (5). Here in the multiorbital spinor, the above interacting Hamiltonian can be collected in a interaction tensor $\tilde{U}_{s/c}$, where the subscripts s and c stand spin and charge density fluctuations, respectively. Of course, it is implicit that all the interaction parameters are orbital dependent. Within the RPA, spin and charge channels become decoupled. The collective many-body corrections of the density-fluctuation spectrum can be written in matrix representation: $\tilde{\chi}_{s/c} = \tilde{\chi}^0[\tilde{1} \mp \tilde{U}_{s/c}\tilde{\chi}^0]^{-1}$, for spin and charge densities, respectively. $\tilde{\chi}^0$ matrix consists of components $\chi^{st}_{0,mn}$ with the same basis in which the interactions $\tilde{U}_{s/c}$ are defined above. Finally, the density fluctuation exchange potentials for the electronic state are computed as

$$V^{st}_{mn,i}(\mathbf{q},\omega_{\mathfrak{p}}) = \frac{\eta_i}{2}\left[\tilde{U}_i\tilde{\chi}''_i(\mathbf{q},\omega_{\mathfrak{p}})\tilde{U}_i\right]^{st}_{mn}, \tag{15}$$

where i stands for spin and charge components, $\eta = 3$, 1 for the spin and charge channels, respectively. The feedback effect of the electronic interaction on the electronic spectrum is then calculated via self-energy calculation within the MRDF method[3–5]

$$\Sigma_{mn,i}(\mathbf{k},\omega) = \frac{1}{\Omega_{\rm BZ}}\sum_{\mathbf{q},st,\nu}\int_{-\infty}^{\infty} d\omega_p V^{st}_{mn,i}(\mathbf{q},\omega_p)\Gamma^{st}_{mn,\nu}(\mathbf{k},\mathbf{q})\left[\frac{1-f^{\nu}_{\mathbf{k-q}}+n_p}{\omega+i\delta-E^{\nu}_{\mathbf{k-q}}-\omega_p}+\frac{f^{\nu}_{\mathbf{k-q}}+n_p}{\omega+i\delta-E^{\nu}_{\mathbf{k-q}}+\omega_p}\right], \tag{16}$$

where the subscript i stands for spin, charge and phonon contributions. The vertex correction $\Gamma^{st}_{mn,\nu}(\mathbf{k},\mathbf{q})$ encodes both the angular and dynamical parts of the vertex, which are combined to obtain $\Gamma^{st}_{mn,\nu}(\mathbf{k},\mathbf{q}) = \phi^{\nu\dagger}_{\mathbf{k-q},s}\phi^{\nu}_{\mathbf{k-q},t}(1 - \partial\Sigma_{mn}(\mathbf{k}-\mathbf{q},\omega)/\partial\omega)_0$. Full self-consistency requires the bare Green's function G_0 in Eq. (12) to be replaced with the self-energy dressed $\tilde{G}^{-1}(\mathbf{k},\omega) = \tilde{G}_0^{-1}(\mathbf{k},\omega) - \tilde{\Sigma}(\mathbf{k},\omega)$, where the total self-energy tensor is $\tilde{\Sigma}(\mathbf{k},\omega) = \tilde{\Sigma}_s(\mathbf{k},\omega) + \tilde{\Sigma}_c(\mathbf{k},\omega) + \tilde{\Sigma}_p(\mathbf{k},\omega)$,y and calculate susceptibilities and self-energies with the dressed Green's function until the self-energies converges. This procedure is numerically expensive, especially in multiband systems and when full momentum dependence is retained. Therefore, we adopt a modified self-consistency scheme, where we expand the real part of the total self-energy tensor as $\tilde{\Sigma}'(\mathbf{k},\omega) = (1-\tilde{Z}_{\mathbf{k}})^{-1}\omega$ in the low-energy region [$|\omega| < 0.2-0.3$ eV in the present materials]. The resulting self-energy dressed quasiparticle dispersions $\bar{\xi}^{\nu}(\mathbf{k}) = Z^{\nu}_{\mathbf{k}}\xi^{\nu}(\mathbf{k})$ are used in Eqs. (12)-(16), which keep all the formalism unchanged with respect to the momentum-resolved orbital-selective quasiparticle renormalization factor $\tilde{Z}_{\mathbf{k}}$.

---

* these authors contributed equally
† milan.radovic@psi.ch

[1] R. S. Dhaka, Tanmoy Das, N. C. Plumb, Z. Ristic, W. Kong, C. E. Matt, N. Xu, K. Dolui, E. Razzoli, M. Medarde, L. Patthey, M. Shi, M. Radovic, J. Mesot, Tuning the metal-insulator transition in $NdNiO_3$ heterostructures via Fermi surface instability and spin fluctuations, Phys. Rev. B **92**, 035127 (2015).

[2] C. Piamonteze, U. Flechsig, S. Rusponi, J. Dreiser, J. Heidler, M. Schmidt, R. Wetter, M. Calvi, T. Schmidt, H. Pruchova, J. Krempasky, C. Quitmann, H. Brune, F. Nolting, X-Treme beamline at SLS: X-ray magnetic circular and linear dichroism at high eld and low temperature, J. Synchrotron Rad. **19**, 661-674 (2012)

[3] T. Das, et al., Adv. Phys. **63**, 151-266 (2014); Phys. Rev. Lett. **108**, 017001 (2012); Phys. Rev. B **91**, 094510 (2015).

[4] T. Das, J. -X. Zhu, and M. J. Graf, Spin-fluctuations and the peak-dip-hump feature in the photoemission spectrum of actinides, Phys. Rev. Lett. **108**, 017001 (2012).

[5] T. Das, and K. Dolui, Superconducting dome in MoS2 and TiSe2 generated by quasiparticle-phonon coupling, Phys. Rev. B **91**, 094510 (2015).

[6] M. P. Seah W. & A. Dench, Quantitative electron spectroscopy of surfaces: A standard data base for electron inelastic mean free paths in solids, Surf. Interface Anal. **1**, (1979).

[7] T. Saitoh, et al., Electronic structure of $La_{1-x}S_xMnO_3$ studied by photoemission and x-ray-absorption spectroscopy, Phys. Rev. B **51**, 13942, (1995).

[8] N. Mannella, et al. Nodal quasiparticle in pseudogapped colossal magnetoresistive manganites. Nature, **438**, 474-478 (2005).

[9] H. Chang, et al., X-ray magnetic circular dichroism and near-edge X-ray absorption fine structure of buried interfacial magnetism measured by using a scanning tunneling microscope tip. Applied Physics Letters bf 113, 061602 (2018).
